# Gut microbiome signatures associated with type 2 diabetes in obesity in Mongolia

**DOI:** 10.3389/fmicb.2024.1355396

**Published:** 2024-06-25

**Authors:** Akari Shinoda, Tsogtbaatar Lkhagvajav, Riko Mishima, Phatthanaphong Therdtatha, Dugersuren Jamiyan, Chantsaldulam Purevdorj, Sainbileg Sonomtseren, Battogtokh Chimeddorj, Bira Namdag, Yuan Kun Lee, Shirchin Demberel, Jiro Nakayama

**Affiliations:** ^1^Division of Systems Bioengineering, Department of Bioscience and Biotechnology, Faculty of Agriculture, Kyushu University, Fukuoka, Japan; ^2^Laboratory of Physiology and Pathology of Young Animals, Institute of Veterinary Medicine, Mongolian University of Life Sciences, Ulaanbaatar, Mongolia; ^3^Division of Biotechnology, School of Agro-Industry, Faculty of Agro-Industry, Chiang Mai University, Chiang Mai, Thailand; ^4^Department of Endocrinology, The First Central Hospital of Mongolia, Ulaanbaatar, Mongolia; ^5^Department of Endocrinology, Mongolian National University of Medical Sciences, Ulaanbaatar, Mongolia; ^6^Department of Microbiology and Infection Prevention Control, Mongolian National University of Medical Sciences, Ulaanbaatar, Mongolia; ^7^Department of the Gastroenterology, Mongolian National University of Medical Sciences, Ulaanbaatar, Mongolia; ^8^Department of Microbiology and Immunology, National University of Singapore, Singapore, Singapore

**Keywords:** gut microbiome, Mongolian, obesity, type 2 diabetes, *Anaerostipes hadrus*, short-chain fatty acid, whole shotgun metagenomics, tauroursodeoxycholic acid

## Abstract

Mongolian people possess a unique dietary habit characterized by high consumption of meat and dairy products and fewer vegetables, resulting in the highest obesity rate in East Asia. Although obesity is a known cause of type 2 diabetes (T2D), the T2D rate is moderate in this population; this is known as the “Mongolian paradox.” Since the gut microbiota plays a key role in energy and metabolic homeostasis as an interface between food and body, we investigated gut microbial factors involved in the prevention of the co-occurrence of T2D with obesity in Mongolians. We compared the gut microbiome and metabolome of Mongolian adults with obesity with T2D (DO: *n* = 31) or without T2D (NDO: *n* = 35). Dysbiotic signatures were found in the gut microbiome of the DO group; lower levels of *Faecalibacterium* and *Anaerostipes* which are known as short-chain fatty acid (SCFA) producers and higher levels of *Methanobrevibacter*, *Desulfovibrio*, and *Solobacterium* which are known to be associated with certain diseases. On the other hand, the NDO group exhibited a higher level of fecal SCFA concentration, particularly acetate. This is consistent with the results of the whole shotgun metagenomic analysis, which revealed a higher relative abundance of SCFA biosynthesis-related genes encoded largely by *Anaerostipes hadrus* in the NDO group. Multiple logistic regression analysis including host demographic parameters indicated that acetate had the highest negative contribution to the onset of T2D. These findings suggest that SCFAs produced by the gut microbial community participate in preventing the development of T2D in obesity in Mongolians.

## 1 Introduction

In Asia, lifestyle-related diseases are known to be associated with social problems. In particular, East Asia, where there have been rapid changes in diet owing to the introduction of an urban lifestyle, is facing an epidemic of metabolic diseases. In Asian people, however, metabolic diseases arise not only from diet but also from phenotypic and genotypic factors (Ramachandran et al., 2012; Mathis et al., 2022). Notably, East Asians tend to have higher abdominal obesity than other ethnic groups (Ramachandran et al., 2012); this is associated with their susceptibility to glucose homeostasis (Hu, 2011; Kodama et al., 2013) and lower pancreatic β-cell mass, which predisposes them to impaired insulin secretion even in relatively lean subjects. In fact, the type 2 diabetes (T2D) risk is higher in lower-body mass index (BMI) Asian populations than in lower-BMI Western populations (Chan et al., 2009).

The gut microbiota plays a key role in metabolic control and homeostasis (Pinart et al., 2022; Zhou et al., 2022). It is reported that the gut microbiome exhibits lower alpha-diversity and a different community structure in obesity than non-obesity people (Therdtatha et al., 2021; Pinart et al., 2022). Gut microbiota dysbiosis, in which reduced gut barrier function enables gut microbes and their components to enter the bloodstream, is associated with chronic diseases, including T2D (Scheithauer et al., 2020; Yang et al., 2021; Mishra et al., 2023). In contrast, microbiota-secreted short-chain fatty acids (SCFAs), specifically acetate, propionate, and butyrate, play important roles in preventing metabolic disorders by maintaining both metabolic homeostasis and the gut barrier, and via their anti-inflammatory activity (Portincasa et al., 2022). Levels of *Roseburia* and *Faecalibacterium*, SCFA-producing bacteria, are reduced in patients with T2D (Martínez-López et al., 2022).

Mongolia, a landlocked East Asian country, exhibits a unique dietary habit characterized by high consumption of animal products and low consumption of plant matter, established traditionally under a nomadic lifestyle adapted to the dry climate (Bromage et al., 2020; Delgermaa et al., 2023). This leads to insufficient dietary intake of particular components, including fiber, folate, and vitamin D, and can lead to obesity (Bromage et al., 2020). In 2016, 20.4% of the population of Mongolia suffered from obesity (BMI > 30) (NCD Risk Factor Collaboration, 2020), the highest prevalence among East Asian countries. However, the incidence of lifestyle-related diseases such as diabetes and coronary heart disease is lower than expected; this is recognized as the “Mongolian paradox” (Torii et al., 2012). This is possibly explained by their unique dietary habit; extremely high consumption of dairy products, particularly fermented animal milk with probiotic effect, and also supplementary consumption of medical plants and herbs with antioxidant effects (Torii et al., 2012).

Gut microbiota of Mongolians has attracted interest in relation to their unique dietary habit. The first study on the gut microbiota of Mongolian people (Zhang et al., 2014) examined regional and seasonal differences, finding that the core intestinal microbiota comprised *Prevotella, Bacteroides,* and *Faecalibacterium*. A comparative study with Han and European populations found that *Faecalibacterium prausnitzii* and *Coprococcus comes* are enriched in Mongolian people which may contribute to their health through the production of SCFA, notably butyrate with anti-inflammatory activity (Liu et al., 2016). Our earlier study (Shinoda et al., 2021) showed that the general Mongolian population has *Prevotella*-dominant microbiome in their gut and that lactic acid bacteria are enriched in Mongolian relative to other Asian populations.

The gut microbiota is now recognized as an interface between foods and host health, notably energy and metabolic homeostasis. In this context, the gut microbiome has to be investigated to address the Mongolian paradox. Therefore, we herein investigated gut microbial factors involved in the prevention of the co-occurrence of T2D with obesity in Mongolians, by comparing the gut microbiome of Mongolian individuals with obesity with and without T2D; first, the difference in the bacterial composition was investigated by the 16S rRNA amplicon sequencing, second, the difference in the metabolites was investigated by targeting short-chain fatty acids (SCFAs) and bile acids (BAs), third, the difference of their community functional potential was assessed by the shotgun metagenomics, and finally we address the alteration in the community function correlated with the development of T2D in Mongolian with obesity.

## 2 Materials and methods

### 2.1 Ethics declaration

This study was approved by the Ethics Committees of the Faculty of Agriculture in Kyushu University (Approval No. 107). Ethical clearance was obtained from the Ethics Committee of the Ministry of Health of Mongolia (Approval No. 78). All methods were performed in accordance with the relevant guidelines and regulations. Written informed consent was obtained from all participants, and the samples and data were analyzed, entered, and published anonymously.

### 2.2 Study design

Participants were Mongolian adults living in Ulaanbaatar (*n* = 39) and Bulgan (*n* = 27). Subjects were screened according to the inclusion and exclusion criteria for AMP phase IV study (published in the Supplementary Material of Therdtatha et al., 2021), except that some of the subjects had received antidiabetic therapy in the past 2 months. Supplementary Table S1 presents details about the subjects.

Samples were collected from the 66 participants with obesity (BMI > 30), who were classified into two groups, with T2D (DO: HbA1c ≥ 6.5) or without T2D (NDO: HbA1c < 6.5), based on the National Glycohemoglobin Standardization Program (NGSP) criteria. Table 1 summarizes the demographic and clinical characteristics of each group. There were significant differences in age, sex, and BMI between the DO and NDO groups; these were subsequently treated as non-microbial confounding factors. The NDO group was further divided into a prediabetes group (PDO, HbA1c > 5.7) and a healthy obese group (HO, HbA1c ≤ 5.7) to examine differences in microbiome community structure.

**Table 1:** Demographic and clinical characteristics of the 66 Mongolian subjects.

	Diabetic obesity (DO)	Non-diabetic obesity (NDO)	*p* value
*N*	31	35	
Gender (male/female)	18/13	10/25	0.0243^a^
Region (rural/urban)	12/19	8/27	0.189^a^
Age (year)	53.0 ± 7.9	49.0 ± 7.7	0.00485^b^
BMI	33.7 ± 4.7	32.0 ± 2.7	0.047^b^
HbA1c (%)	8.1 ± 3.0	5.7 ± 0.35	<0.001^b^
Fasting blood glucose*	201.6 ± 73.0	111.0 ± 8.2	<0.001^b^
Anti-diabetic drugs (no.)	11	0	

a Significant difference between the obese (DO) and nondiabetic obese (NDO) groups (Fisher’s exact test).

b Significant difference between the DO and NDO groups (Wilcoxon rank-sum test).

*Information missing for three subjects in the DO group.

### 2.3 Sample collection

All subjects provided two fresh stool samples for metagenome and metabolome analyses, in separate tubes (76 × 20 mm, Sarstedt, Munich, Germany), each containing 2 mL RNAlater (Invitrogen, Thermo Fisher Scientific, Waltham, MA) or MeOH, and five YTZ 2.5 mm zirconia balls (Nikkato, Sakai, Japan). For each tube, two spoons of fecal sample were collected from different parts in the middle section of feces and immediately suspended in the solution by shaking the tubes vigorously.

The collected feces were transferred to the laboratory, vortexed to completely suspend the feces, and stored at −80°C in Mongolian University of Life Sciences. The samples were transported to Kyushu University, Japan, while kept below 4°C and were immediately stored at −80°C until DNA or metabolite extraction.

### 2.4 DNA extraction, 16S rRNA gene amplicon sequencing, and data processing

Total bacterial DNA was extracted from stool samples using the bead-beating method, as previously described (Matsuki et al., 2004). Briefly, DNA was extracted by first vigorously shaking the feces with the zirconium beads; the DNA was then purified by phenol–chloroform extraction followed by isopropanol precipitation (Supplementary Material of Therdtatha et al., 2021). High-throughput 16S rRNA gene sequencing was performed as previously described (Kisuse et al., 2018). Briefly, the V3 to V4 region of the bacterial 16S rRNA gene was amplified via two-step PCR, in which the bacterial universal primer set, Bakt_341F (5′-CGCTCTTCCGATCTCTGCCTACGGGNGGCWGC AG-3′) and Bakt_805R (5′-TGCTCTTCCGATCTGACGACTA CHVGGGTATCTAATCC-3′), was used in the first step; in step two, the same primers plus index-sequences were used. Amplicons with index sequences were subjected to sequencing using the MiSeq Reagent Kit v3 (MS-102-3003; Illumina, San Diego, CA, United States).

The sequence data were processed using QIIME2 pipeline 2021.2 (Bolyen et al., 2019). Briefly, after the total reads were demultiplexed using the index sequences, paired-end sequences were merged and denoised, and primer sequences were removed using DADA2 (Callahan et al., 2016). The forward and reverse primers were eliminated using the 3′ and 5′ end trim parameters “--p-trim-left-f 17” and “--p-trim-left-r 21,” respectively. The parameters “p-trunc-len-f 290” and “p-trunc-len-r 210” were used to trim the forward and reverse reads to lengths of 290 and 210 bases, respectively. The sequence of each amplicon sequence variant (ASV) with 97% sequence identity was taxonomically classified via the QIIME “classify-sklearn” feature using the SILVA 138 database.

### 2.5 Beta-diversity analysis

Beta diversity was assessed using Bray Curtis, Jaccard, weighted UniFrac, and unweighted UniFrac distances, and permutational multivariate analysis of variance (PERMANOVA) was used to investigate statistical differences between the PDO and HO groups and between the DO and NDO groups.

### 2.6 Whole shotgun metagenomics

For whole shotgun metagenomic analysis, nine samples were selected from both the DO and NDO groups to be evenly distributed in the weighted and unweighted UniFrac-PCoA ordinations of both groups (Supplementary Figure S1). The DNA extract prepared for the 16S rRNA amplicon sequencing was treated using RNase (NIPPON GENE Co., Ltd., Tokyo, Japan) and was purified using a Gel/PCR extraction kit (NIPPON GENE Co., Ltd., Tokyo, Japan) to remove RNA. DNA concentration was quantified using the Quant-iT PicoGreen dsDNA Assay Kits with a dsDNA standard (Invitrogen, Thermo Fisher Scientific, Waltham, MA). More than 1 μg of DNA in TE buffer (>12.5 ng/μL) was subjected to 150 bp paired-end sequencing on a DNBSEQ system (BGI, Shenzhen, China).

The obtained raw sequences were filtered using the SOAPnuke tool (Chen et al., 2018) to remove (i) reads matching ≥25.0% of the adapter sequences (up to three base mismatches allowed); (ii) short reads (<150 bp); (iii) reads in which undetermined nucleotides account for ≥0.1% of the entire read; and (iv) low-quality reads in which bases with quality <20 accounts for ≥40% of the entire read. Then, the cleaned pair-ended reads were merged by using BBMerge (Bushnell et al., 2017). Human DNA sequences were then removed using the KneadData tool and the database Homo_sapiens_hg37_and_human_contamination_Bowtie2_v0.1. The sequences were further processed using the KneadData tool (version 0.12.0) with the default settings and the human DNA sequences were removed by Bowtie2 (version 2.4.2) on very-sensitive-local mode, phred33 parameter, and the database Homo_sapiens_hg37_and_human_contamination_Bowtie2_v0.1.

These quality-filtered sequences were functionally profiled using the HUMAnN pipeline (v. 3.6.1) using MetaPhlAn 4.0; the mpa_vJan21_CHOCOPhlAnSGB_202103 nucleotide database was used as an intermediate step in the taxonomic classification of each DNA fragment. Each DNA fragment was functionally annotated to UniRef90 gene families, and then categorized into Kyoto Encyclopedia of Genes and Genomes (KEGG) categories at the enzyme, orthology, and pathway levels. Gene abundance in each sample (and similarly in each category) was calculated as reads per kilobase (RPK) and was normalized to obtain the count per million (CPM).

### 2.7 Quantification of SCFAs and bile acids

SCFAs and BAs in the feces were quantified as previously described (Tanaka et al., 2020; Therdtatha et al., 2021). Briefly, feces collected in MeOH were placed in a SpeedVac concentrator (Thermo Fisher Scientific, Waltham, MA) to evaporate the methanol, and were resuspended in PBS in deuterated water (100 mM, pH 7.4) containing 3-(trimethylsilyl) propionate-2.2.3.3-*d*_4_ as an internal standard. The supernatant was used for quantitative 1-H nuclear magnetic resonance (NMR) spectroscopy to quantify acetate, propionate, and butyrate, using a 400 MHz spectrometer (JNM-ECZ400, JEOL, Tokyo, Japan). The SCFA data of each sample was deposited in Supplementary Table S3.

BAs were extracted from the fecal pellet by hot ethanol with an internal standard of nor-deoxycholic acid at 60°C for 30 min and subsequently at 100°C for 3 min. After cleaning using an Oasis HLB cartridge column (WAT094225, Waters, Milford, MA), the extract was subjected to liquid chromatography with a triple quadrupole mass spectrometry system, in which 15 major human fecal BAs were quantified (LCMS-8050, Shimadzu, Japan). These procedures are described in detail by Tanaka et al. (2020). The BA data of each sample was deposited in Supplementary Table S4.

### 2.8 Statistical analysis

Statistical analyses and data visualization were performed using RStudio 2022.07.2, R 4.2.1 (R Core Team, 2023), Stata/SE 12.0 (Stata Technical Support, 2023), and EZR 4.0.3 (Kanda, 2013).

To evaluate statistical differences between two groups, the Wilcoxon rank-sum test was applied using “wilcox.test” in R. To evaluate statistical differences in qualitative variables between groups, Fisher’s exact test was used in EZR. Spearman’s correlation analysis was performed to calculate correlations between indices, using Stata/SE12. Multiple logistic regression analysis was performed using EZR to assess the risk of each factor to the occurrence of T2D. Linear discriminant analysis of effect size (LEfSe) was performed using the online Galaxy platform (Segata et al., 2011).

### 2.9 Accessions of sequence data

The raw sequence data obtained was deposited in the DNA Data Bank of Japan (DDBJ) (Fukuda et al., 2021) under BioProject no. PRJDB5860. The Sequence read archives of 16S rRNA gene amplicon sequences (DRA017379) and whole shotgun metagenomic sequence data (DRA017451) were deposited with fecal sampling data in the BioSample database (BioSample, 2023) (accessions SAMD00656652-717 and SAMD00657900-17, respectively).

## 3 Results

### 3.1 Differences in the microbial community composition between the DO and NDO groups

The gut microbial community composition was profiled by barcode sequencing of 16S rRNA V3-V4 region. Eventually, 812,093 high quality sequences corresponding to 12,304 ± 2,774 reads (minimum 6,231) per sample were obtained from 17,273 ± 3,757 raw reads per sample and were clustered into 1,722 ASVs; these were assigned to 2 domains, 13 phyla, 19 classes, 40 orders, 72 families, 218 genera, and 502 species. The counts of each ASV for each sample were tabulated in Supplementary Table S2 with taxonomic information for each ASV.

Differences in gut microbial composition were calculated using four distance matrices, and discreteness between the different diabetic status groups was examined using permutation analysis (Table 2). The DO and NDO groups differed significantly, based on the Jaccard, Bray-Curtis, and unweighted UniFrac distances (Supplementary Figure S1 for the ordination plot). When the NDO group was further divided into PDO and HO groups only the Jaccard distance showed a significant difference between DO and PDO, while there were no significant differences between the PDO and HO groups. This indicates that the DO group harbored a significantly different gut microbial community from the NDO group, whereas the community did not differ significantly between the PDO and HO groups. Therefore, the grouping of DO and NDO including PDO and HO subjects was henceforth used in this study.

**Table 2:** Pairwise differences in microbial community composition, based on Jaccard, Bray-Curtis, weighted UniFrac, and unweighted UniFrac distances.

Pairwise adonis				Jaccard	Bray-curtis	Weighted UniFrac	Unweighted UniFrac
Three groups	DO	vs	PDO	**0.039**	0.753	1	0.081
	DO	vs	HO	0.108	0.129	0.078	0.441
	PDO	vs	HO	1	1	0.291	1
Two groups	DO	vs	NDO	**0.002**	**0.02**	0.31	**0.014**

Statistical significance between groups was assessed by PERANOVA (pairwise adonis). Values in bold: significant differences at *p* < 0.05.

**Figure 1:**
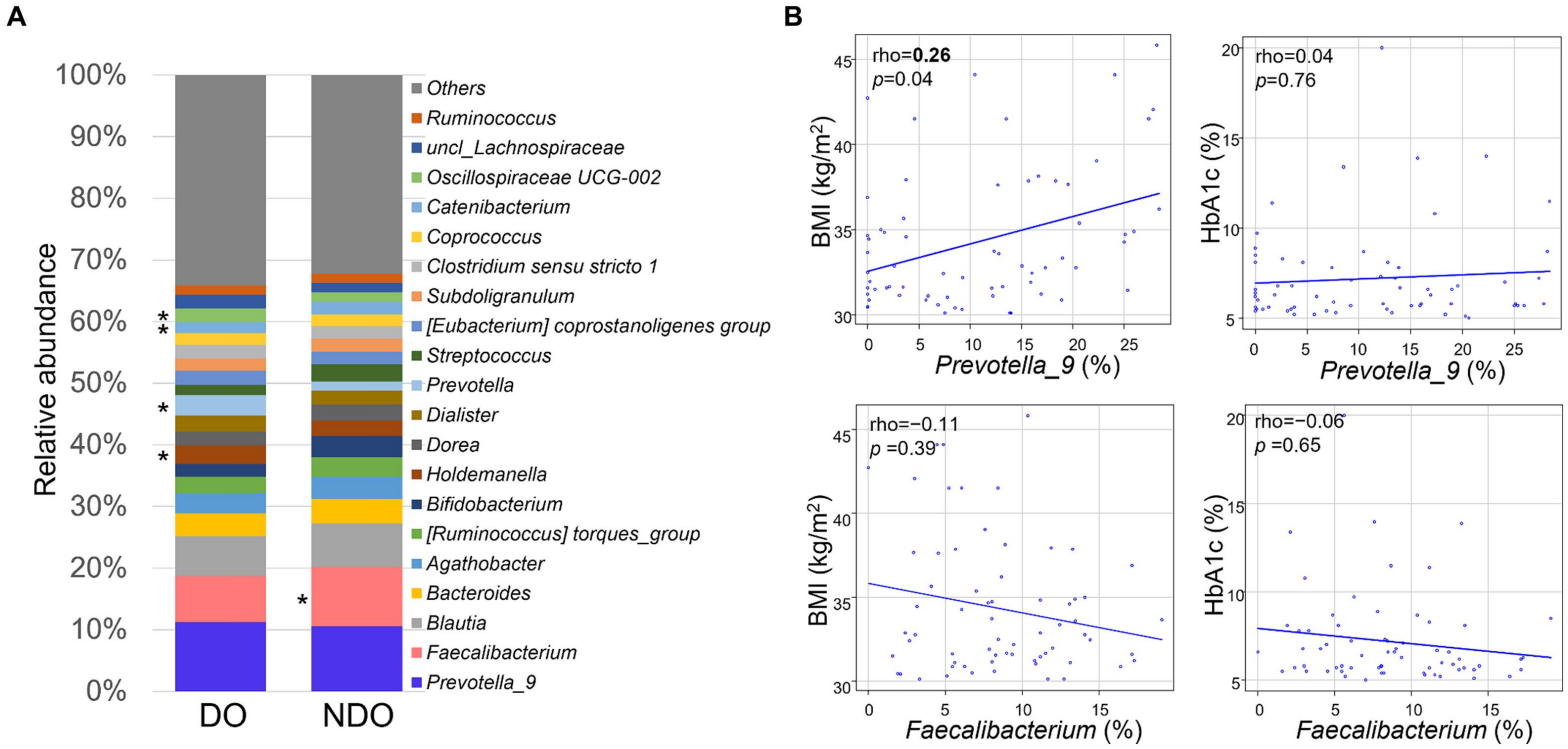
Composition and abundance of the dominant fecal bacterial genera in fecal samples from Mongolian subjects with obesity. **(A)** Comparison of the top 20 genera in the diabetic obese (DO) and nondiabetic obese (NDO) groups. The abundance of each genus is shown in the bar graph. *, higher values in the relevant group, approaching significance (*p* < 0.1). **(B)** Correlations between the abundance of *Prevotella*_9 or *Faecalibacterium* and BMI or HbA1c levels. Correlations were analyzed using Spearman’s rank sum test.

The relative abundances of the 20 most abundant genera are illustrated separately for the DO and NDO groups (Figure 1A). In both groups, the most abundant genus was *Prevotella_9*, followed by *Faecalibacterium* and *Blautia*. Although there was no significant difference in the abundances of the top 20 genera between the DO and NDO groups, some trends were observed, including elevated *Faecalibacterium* (*p* = 0.066) levels and lower *Holdemanella* (*p* = 0.089) levels in the NDO group. Correlations between the abundance of the most or second most dominant genera (*Prevotella_9* and *Faecalibacterium*, respectively) and BMI or HbA1c were calculated using the Spearman’s rank sum test (Figure 1B); *Prevotella*_9 was significantly positively correlated with BMI.

**Figure 2:**
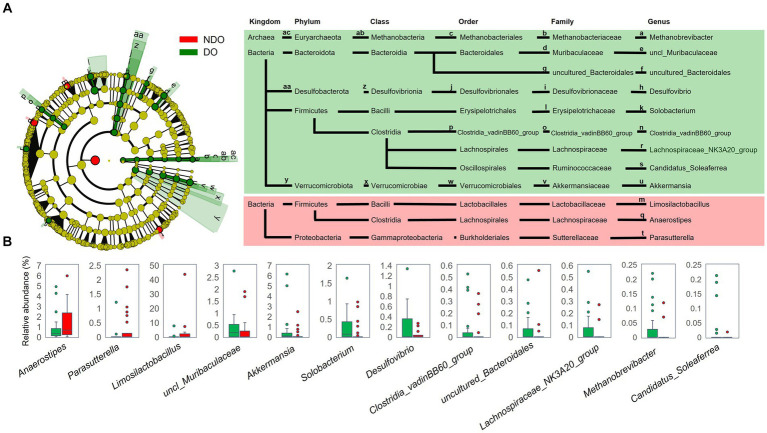
Comparison of fecal bacterial composition between the diabetic obese (DO; green) and nondiabetic obese (NDO; red) groups. **(A)** Linear discriminant analysis of effect size (LEfSe) analysis to compare the fecal bacterial composition at each taxonomic rank between the DO and NDO groups. The Wilcoxon rank-sum test was used to calculate the linear discriminant analysis (LDA) scores. Taxonomic groups with LDA > 2.0 and *p* < 0.05 are highlighted by the indicated color on the cladogram. **(B)** Box plots of the abundance of genera that differed significantly between the DO and NDO groups in the LEfSe analysis.

LEfSe analysis was performed to examine differences in gut microbiota composition at different taxonomic levels between the DO and NDO groups (Figure 2A): the genera *Anaerostipes*, *Limosilactobacillus* and *Parasutterella* were significantly abundant in the NDO group, whereas in the DO group, a wide variety of taxonomic groups belonging to domain Archaea, six bacterial families, and nine bacterial genera, including *Methanobrevibacter*, *Desulfovibrio*, and *Solobacterium*, were significantly abundant (Figure 2B). Further, correlation analysis (Supplementary Table S5) indicated that *Methanobrevibacter* and *Solobacterium* positively correlated with HbA1c.

### 3.2 Comparison of fecal SCFA levels between the DO and NDO groups

**Figure 3:**
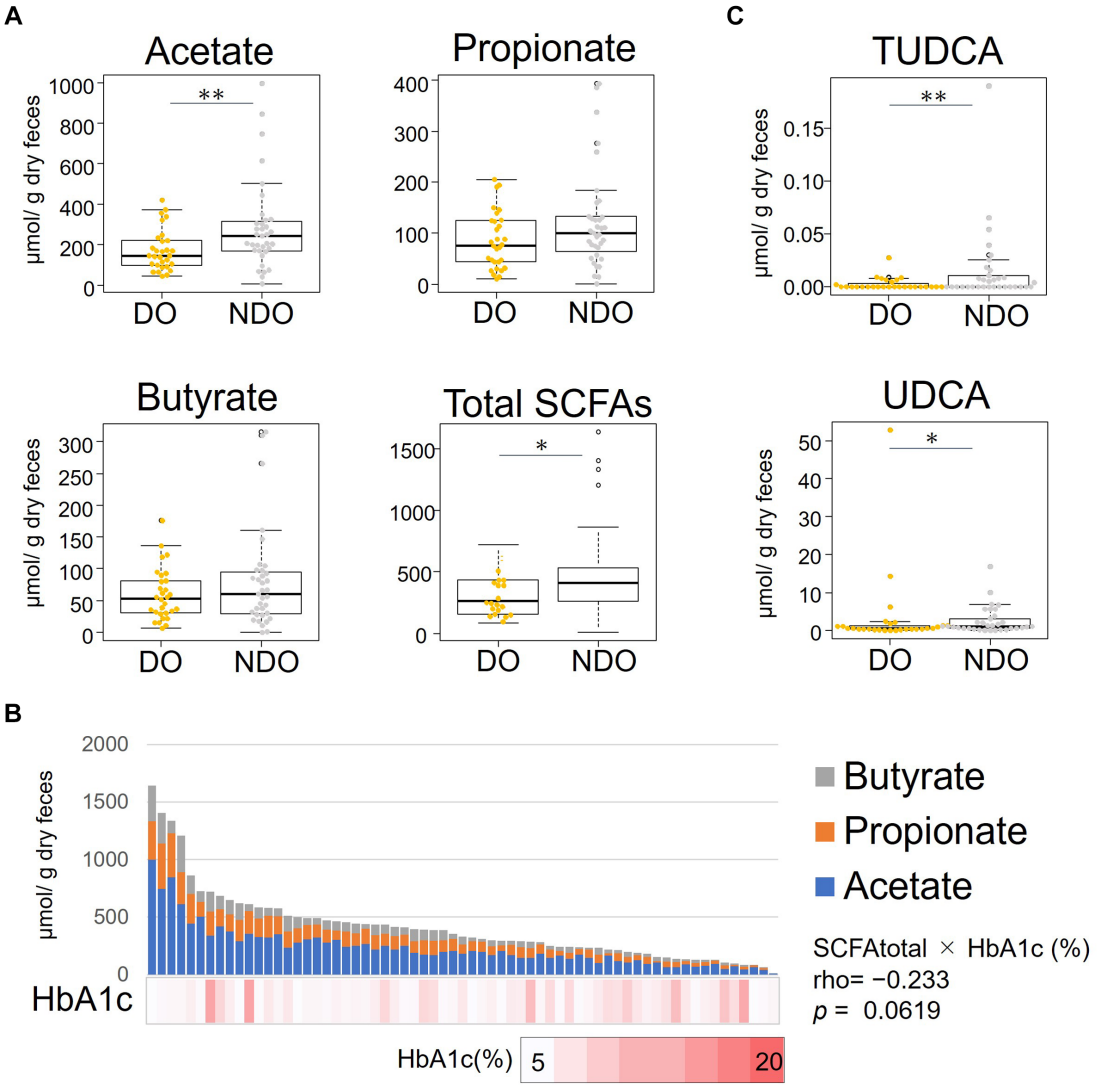
Distribution of the abundances of short-chain fatty acids (SCFAs) and bile acids (BA) in the feces of the diabetic obese (DO) and nondiabetic obese (NDO) groups. **(A)** Comparison of fecal concentrations of acetate, propionate, butyrate and of total SCFA (sum of acetate, propionate, and butyrate). The box plot illustrates these levels in the DO and NDO groups; statistical significance was evaluated using the Wilcoxon rank-sum test. *, 0.05 < *p* < 0.1, **, *p* ≤ 0.05. **(B)** Stacked bar plot showing the levels of the three SCFAs, order by total abundance, in each of the 66 Mongolian subjects. The HbA1c level of each subject is shown in the heatmap below the bar graph. Correlations between the levels of total SCFA and HbA1c were analyzed using Spearman’s rank sum test. **(C)** Comparison of fecal concentrations of tauroursodeoxycholic acid and ursodeoxycholic acid. Statistical differences between groups were calculated as in **(A)**. *, 0.05 < *p* < 0.1, **, *p* ≤ 0.05.

We compared fecal SCFA concentrations between the DO and NDO groups (Figure 3A). The levels of the three major SCFAs–acetate, propionate, and butyrate–tended to be higher in the NDO group, and acetate levels were significantly higher in the NDO group. Figure 3B illustrates the abundance of each SCFA stacked in the bar graph and ordered according to total SCFA concentration, together with a heat map of HbA1c levels (Figure 3B). In most samples, the ratio of acetate, propionate, and butyrate was approximately 6:3:2. The Spearman’s rank sum test revealed an inverse correlation between total SCFA concentration and HbA1c with approached significance (*r* = −0.23, *p* = 0.06).

### 3.3 Comparison of fecal BA levels between the DO and NDO groups

We measured the concentrations of 15 major BAs in fecal samples (Figure 3C). Ursodeoxycholic acid and its taurine conjugated forms, tauroursodeoxycholic acid (TUDCA), tended to be more abundant in the NDO group. In particular, levels of TUDCA were significantly higher in the NDO group than in the DO group (*p* = 0.049).

### 3.4 Comparison of metagenomic profile between the DO and NDO groups

**Figure 4:**
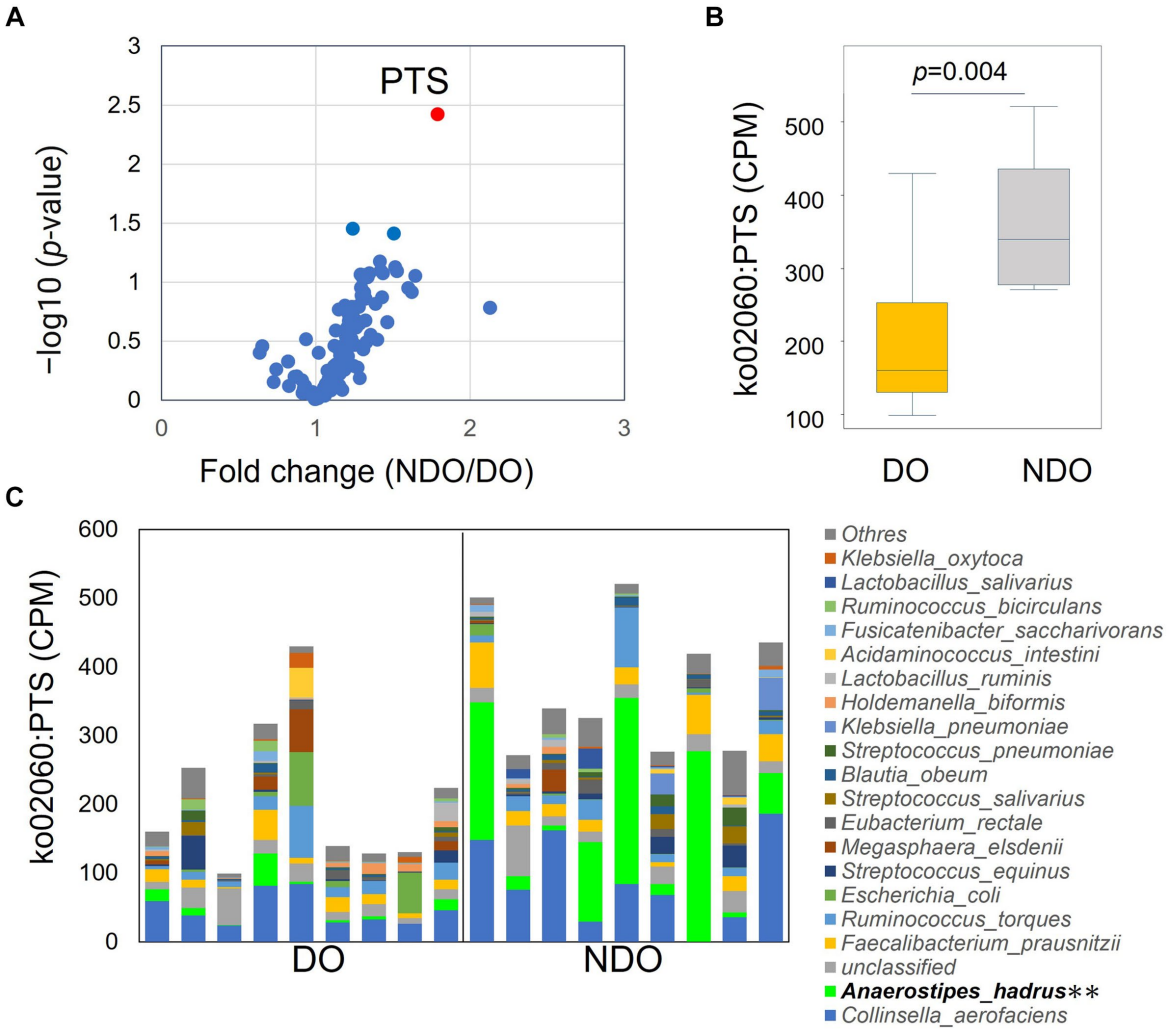
KEGG pathway differential enrichment in the diabetic obese (DO) and nondiabetic obese (NDO) groups. **(A)** Volcano plot illustrating differences in the abundance of each KEGG pathway between the DO and NDO groups. The vertical axis displays –log_10_*p* and the horizontal axis displays the linear fold change (NDO/DO). **(B)** Box plot of ko02060 (PTS) expression (in count per million, CPM) in the DO and NDO groups. **(C)** Stacked bar plot of ko02060 (PTS) expression (in count per million, CPM) in each species. **p < 0.05.

**Figure 5:**
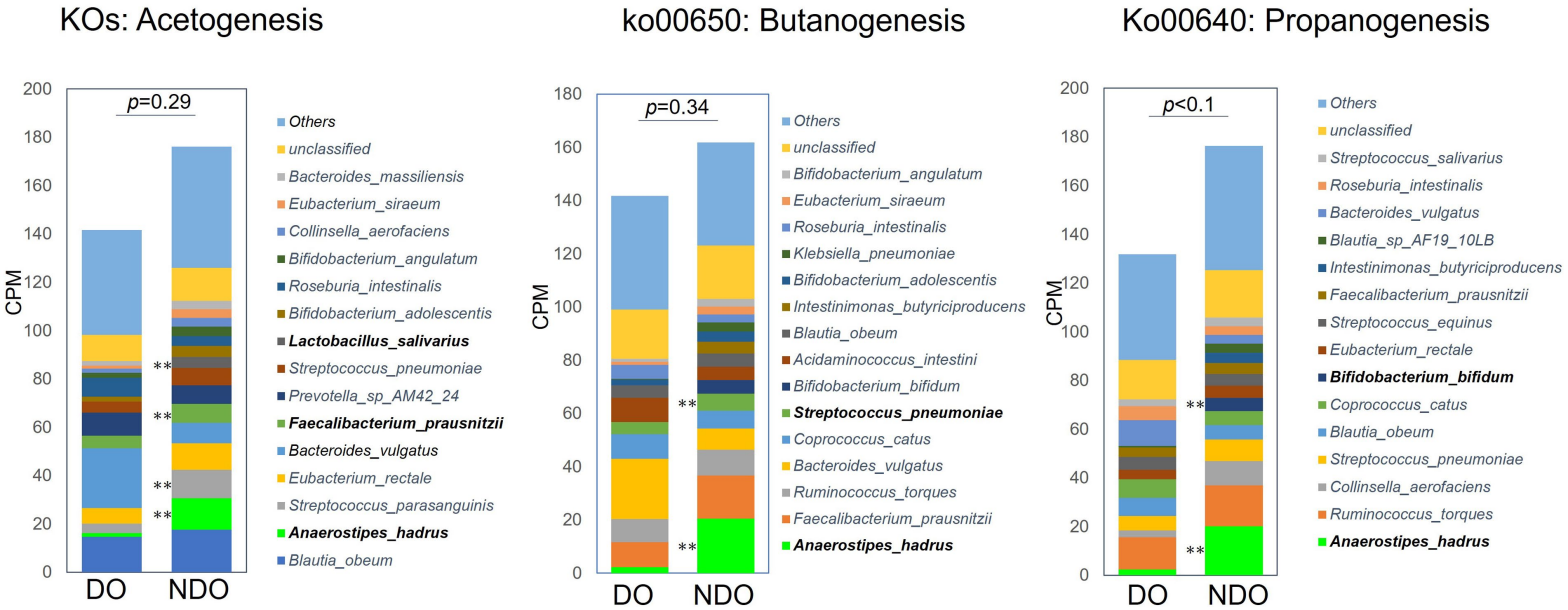
Abundances of KEGG pathway/orthology involved in short-chain fatty acid (SCFA) biosynthesis in the diabetic obese (DO) and nondiabetic obese (NDO) groups. The three stacked bar plots refer, respectively, to the KEGG pathways/orthologies involved in acetogenesis, propanogenesis (ko00640), and butanogenesis (ko00650), and reflect count per million (CPM). For acetogenesis, the counts of KEGG orthologies involved in the Wood–Ljungdahl pathway (17 KOs) and of the K00158 (pyruvate oxidase) and K01512 (acyl phosphatase) KOs, were summed. **p < 0.05 comparing the DO and NDO groups.

To address differences in the functional potential of the microbial communities in the DO and NDO groups, we performed a whole shotgun metagenomic analysis by using 9 DO and 9 NDO samples. Ultimately, per sample, 24.01 ± 2.48 million high-quality non-human reads, corresponding to 5.60 ± 0.48 Gb sequences, were obtained from 24.30 ± 2.50 million raw reads corresponding to 5.64 ± 0.48 Gb sequences. In the KEGG pathway categories, only the phosphotransferase system (PTS) exhibited significantly differential enrichment (Figures 4A,B). The enrichment of PTS genes in the NDO group can be attributed to the marked enrichment of *Anaerostipes hadrus* (Figure 4C). Further, genes involved in SCFA biosynthesis tended to be more abundant (although not significantly) in the NDO group, with *A. hadrus* contributing to this enrichment (Figure 5).

**Figure 6:**
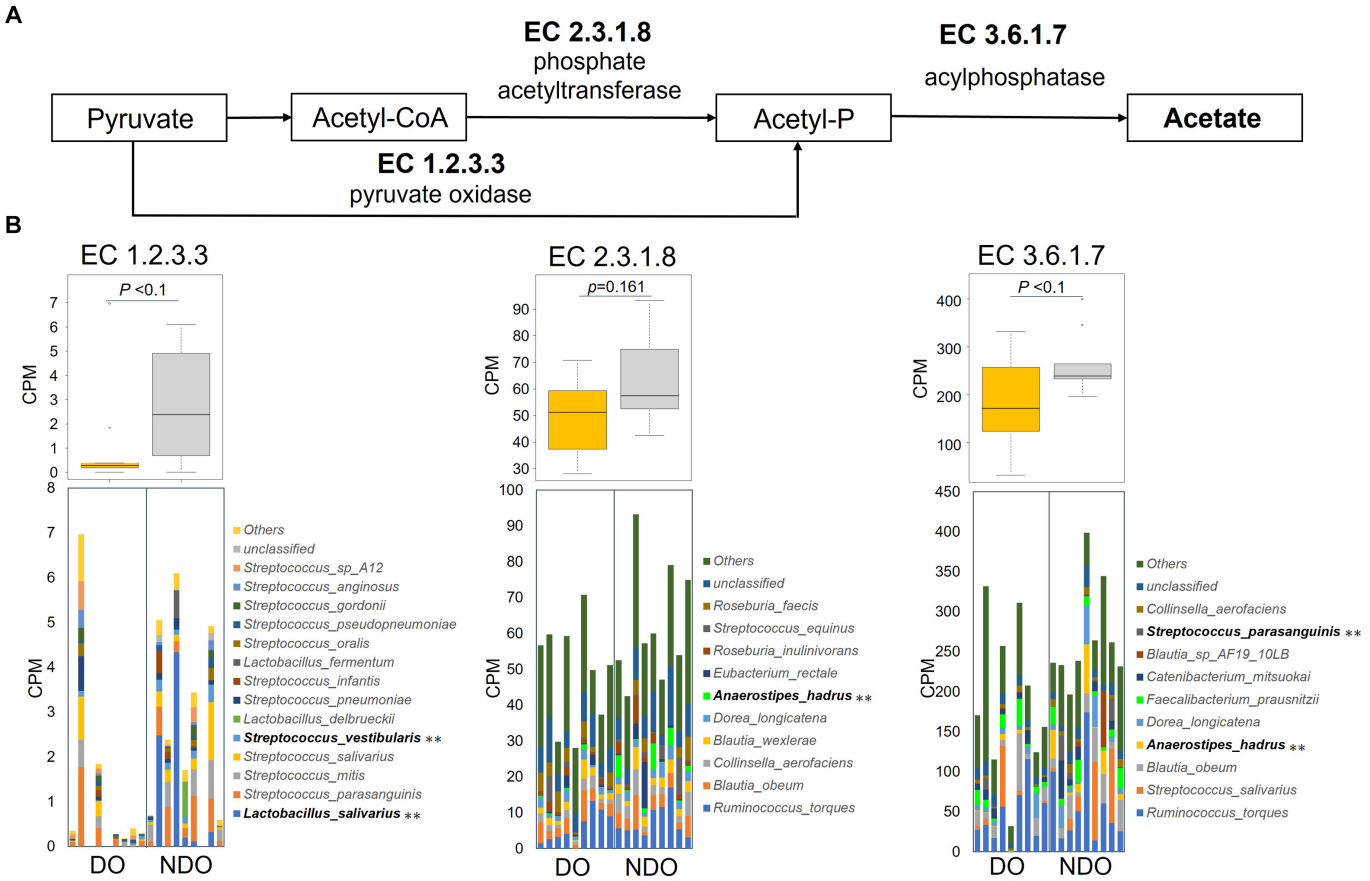
Abundances of KEGG enzymes involved in acetogenesis in the diabetic obese (DO) and nondiabetic obese (NDO) groups. **(A)** KEGG enzyme map for the acetogenesis pathway from pyruvate to acetate. **(B)** Box plot showing the counts of each acetogenesis KEGG enzyme (EC1.2.3.3, pyruvate oxidase; EC2.3.1.8, phosphate acetyltransferase; and EC3.6.1.7, acylphosphatase), and stacked bar plots showing the counts of KEGG enzymes per species per subject. **p < 0.05 comparing the DO and NDO groups.

A series of genes encoding enzymes involved in acetogenesis, pyruvate oxidase (EC 1.2.3.3), phosphate acetyltransferase (EC 2.3.1.8), and acylphosphatase (EC 3.6.1.7), were higher in the NDO group than in the DO group (Figures 6A,B), although nonsignificantly. Figure 6B illustrates the composition of the bacteria encoding the enzymes (based on KEGG database) in each group. The enrichment of pyruvate oxidase (EC 1.2.3.3), which metabolizes acetyl phosphate from pyruvate, was attributed to the higher abundance of oral lactic acid bacteria (*Lactobacillus* and *Streptococcus* species) in the NDO group. The higher abundance of *A. hadrus* was responsible for the enrichment in the NDO group of phosphate acetyltransferase (EC 2.3.1.8) and acylphosphatase (EC 3.6.1.7) which consecutively metabolize acetyl-CoA to acetate.

### 3.5 Multiple logistic regression of factors associated with the occurrence of T2D in obesity

To examine the effect of fecal SCFA levels and host demographic factors on the occurrence of T2D in obesity in this population, we performed multiple logistic regression analyses. Model 1A and Model 1B were obtained with all sets of possible host demographic confounding factors and the fecal concentration of either acetate or total SCFA (acetate + propionate + butyrate), respectively, as microbial factors (Table 3). This model predicted the occurrence of T2D with statistical significance; acetate and total SCFA contributed the most respectively, and age and BMI exhibited significant correlations, whereas sampling region, sex, and metformin intake did not. Regarding sampling region, we further investigated the differences in the levels of acetate and total SCFA in Ulaanbaatar and Bulgan, separately, and confirmed their higher levels in both regions, although not statistically significant (Supplementary Figure S2). Regarding metformin, it is known that metformin intake increases the level of SCFA (Mueller et al., 2021). However, we observed an opposite trend that the acetate and total SCFA levels decreased in the metformin-prescribed subjects in the DO group (DO-Met (+)) (Supplementary Figure S3). The DO-Met (+) group showed significantly higher BMI, HbA1c and fasting blood glucose levels than the non-metformin-prescribed subjects in the DO group (DO-Met (−)). The fact that the DO-Met (+) group had significantly more sever diabetic symptom appears to cause the decrease of the SCFA level rather than the direct effect by metformin.

**Table 3:** Model 1 of multiple logistic regression analysis of factors potentially associated with the occurrence of T2D in obesity in the Mongolian subjects.

Factors	Adjusted odds ratio (95% CI)	*p* value	Additional *R*^2^ contribution
Model 1A (*p* = 0.0002, pseudo *R*^2^ = 0.3777)			
**Acetate (100 mmol/g feces)**	**0.41 (0.178–0.946)**	0.037*	**0.15**
Age (y)	1.17 (1.02–1.34)	0.022*	0.118
BMI (kg/m^2^)	1.29 (1.03–1.62)	0.029*	0.094
Region (rural = 0, urban = 1)	0.412 (0.0672–2.53)	0.338	0.014
Gender (female = 0, male = 1)	3.44 (0.677–17.5)	0.136	0.036
Metformin (no = 0, yes = 1)	4.0.E+08	0.994	−0.012
(Intercept)	0.000 (0.000–0.004)	0.003	
Model 1B (*p* = 0.0003, pseudo *R*^2^ = 0.355)			
**Total SCFAs (100 mmol/g feces)**	**0.673 (0.457–0.992)**	0.045*	**0.128**
Age (y)	1.16 (1.02–1.33)	0.024*	0.115
BMI (kg/m^2^)	1.28 (1.03–1.59)	0.029*	0.092
Region (rural = 0, urban = 1)	0.357 (0.061–2.09)	0.254	0.02
Gender (female = 0, male = 1)	3.53 (0.714–17.5)	0.122	0.039
Metformin (no = 0, yes = 1)	2.57.E+08	0.994	−0.025
(Intercept)	0.000 (0.000–0.004)	0.002	

Asterisks represent statistically significance (*p* < 0.05). The bold values indicate the results of the multiple logistic regression analysis for acetate and total SCFAs.

**Figure 7:**
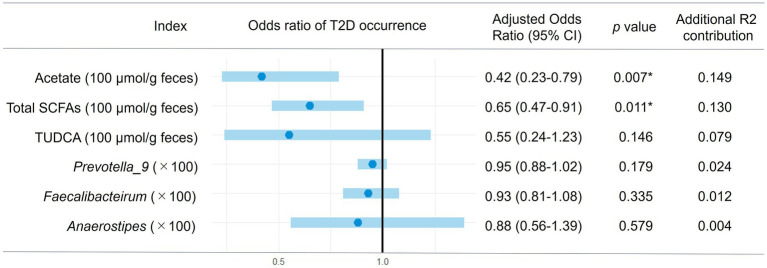
Multiple logistic regression of gut microbial factors for the occurrence of T2D in obesity. The multiple logistic regression analysis of each gut microbial factor was performed with age and BMI (Model 2) and the estimated 95% confidence interval for odds ratio of each factor are shown in the forest plot. Asterisks represent statistically significance (*p* < 0.05).

Following Model 1, we did the multiple logistic regression analysis excluding these insignificant valuables, resulting in Model 2 with enhanced significance and reasonably high prediction strength (*p* < 0.0001 and pseudo *R*^2^ = 0.3285 for the model including acetate and *p* < 0.0001 and pseudo *R*^2^ = 0.3091 for the model including total SCFA). As shown in Figure 7, the odds ratios of acetate and total SCFA were −0.42 and −0.65 per 100 μmol/g feces, respectively. By using this model, we further examined the odds ratios of the abundances of the certain bacteria genera and TUDCA. *Prevotella*_9, *Faecalibacterium*, *Anaerostipes*, and TUDCA showed the tendency of the negative risk associations for the occurrence of T2D, although nonsignificantly.

## 4 Discussion

The main aim of this study was to address the gap of the lower-than-expected frequency of diabetes in obesity in the Mongolian population in terms of intestinal microbiology. First of all, we overviewed the difference in the fecal microbiome structure between the DO and NDO subjects and also between PDO and HO subjects to clarify how the intestinal microbial community differ by the level of diabetes. Community structure differed between the DO and NDO groups, but not between the PDO and HO groups. Various studies have reported significant alterations in the gut microbiota of patients with prediabetes (Letchumanan et al., 2022; Rathi et al., 2023). Our current analysis may have missed small-scale alterations unique to the PDO group, owing to the small sample size. However, the community composition of the NDO group including PDO subjects was clearly different from that of the DO group (Table 2). These differences might be involved in the prevention of T2D development in obesity in Mongolia and were further analyzed.

*Prevotella_9* was the most dominant genus in both DO and NDO groups. Although *Prevotella_9* did not differ in abundance between the DO and NDO groups, it showed positive correlation with BMI (Figure 1B) and also showed marginally lower risk associations with the occurrence of T2D in the multiple logistic regression analysis (Figure 7). This is consistent with prior findings showing a link between obesity and the abundance of *Prevotella*, which exhibits substantial potential to produce energy via high levels of sugar fermentation activity (Precup and Vodnar, 2019). *Prevotella* is also known to exert an antidiabetic function (Kovatcheva-Datchary et al., 2015). Therefore, high levels of *Prevotella* may be involved in the low frequency of T2D in obesity in the Mongolian population.

The second dominant genus, *Faecalibacterium*, was less abundant in the DO group with approached significance (Figure 1A). Lower *Faecalibacterium* abundance is commonly observed in individuals with diabetes (Gurung et al., 2020). A reduction in *Faecalibacterium prausnitzii* abundance was observed even at the prediabetic stage (Wu et al., 2020). Since *Faecalibacterium prausnitzii* is recognized as beneficial, notably as a butyrate producer, its reduction represents dysbiosis and this might be the case in the DO group.

Dysbiotic trend in the DO subjects was also observed in the increase of subdominant genera such as *Methanobrevibacter*, *Desulfovibrio*, and *Solobacterium* (Figure 2). *Methanobrevibacter* is known to induce inflammation by activating dendritic cells to produce inflammatory cytokines (Bang et al., 2014) and its increase has been observed in patients with inflammatory bowel disease (Ghoshal et al., 2016; Ghavami et al., 2018). *Desulfovibrio*, a sulfate-reducing bacterium enriched in T2D subjects (Wang et al., 2012) and in women with gestational diabetes mellitus (Crusell et al., 2018), is generally associated with poor host health (Goldstein et al., 2003). T2D patients who consume higher amounts of dietary fiber exhibited reduced levels of *Desulfovibrio* with reduction of HbA1c (Fu et al., 2022). *Solobacteirum* known as a septicemic infectional bacterium (Detry et al., 2006) is reported to associate with colon cancer (Avuthu and Guda, 2022). These dysbiotic features observed in the DO subjects appear to link with the occurrence of T2D with obesity in Mongolians.

On the other hand, in NDO fecal samples, anti-inflammatory and antidiabetic metabolites, namely TUDCA and SCFAs were found to be elevated (Figure 3). TUDCA acts as an agonist of Takeda G-protein-coupled receptor 5 (TGR5) (Song et al., 2022) and an antagonist of farnesoid X receptor (FXR) (Sun et al., 2018) and thereby shows an anti-inflammatory effect and promotes insulin sensitivity, respectively. The acetate and total SCFA fulfilled statistical significance, even after adjusting for potential confounding variables via multiple logistic regression analysis. The estimated odds ratios of acetate (−0.42 per 100 μmol/g feces) and total SCFA (−0.65 per 100 μmol/g feces) (Figure 7) suggest that increasing their gut levels within the physiological range could have a substantial preventative impact on the development of T2D. The antidiabetic efficacy of SCFAs has been demonstrated in other cohort studies (Zhao et al., 2019, 2020) and in animal model research (Huang et al., 2023). Obesity induces insulin resistance by causing the secretion of proinflammatory cytokines from adipose tissue and via oxidative-stress-induced β-cell dysfunction, resulting in the development of diabetes (Al-Goblan et al., 2014). SCFAs promote the release of glucagon-like peptide 1 (GLP-1) from L cells by activating the epithelial cell receptors FFAR2 (GPR43) and FFAR3 (GPR41), thereby improving insulin sensitivity and secretion (Brown et al., 2003; Kimura et al., 2013; Kaku, 2020). On the other hand, there is an animal study showing that acetate induces metabolic syndrome with hyperphagia through the activation of parasympathetic nervous system (Perry et al., 2016). In our Mongolian subjects, acetate may have these two aspects which promote obesity but protect β-cell dysfunction. Acetate can also be a precursor of acetyl-CoA and subsequently involved in the TCA cycle contribute to glucose synthesis. This kind of gluconeogenesis occurs during fasting conditions and plays a role in thermogenesis, especially under the low-glucose or ketogenic conditions, and is crucially required for avoid of life-threatening hypoglycemia and survival (Gray et al., 2007; Sakakibara et al., 2009). This might be an option equipped in Mongolian who have survived under the nomadic life with severe winter during their long history.

The elevated production of SCFA in the DO group was further demonstrated using whole shotgun metagenomic analysis. Genes for enzymes involved in the SCFA biosynthesis were enriched in the NDO samples, along with the enrichment of PTS genes which participate in carbohydrate uptake (Figure 4) (Kuang et al., 2017; Takeuchi et al., 2023), suggesting higher catabolic potential of gut microbial community in NDO subjects. The metagenomic results identified *A. hadrus* as the primary source of these SCFA-synthesizing enzymes (Figure 5). Indeed, *A. hadrus* is known as acetate, propionate, and butyrate producer equipped with high potential for sugar catabolism with high content of genes encoding phosphotransferase system (Low et al., 2023). A lower abundance of *Anaerostipes* in individuals with diabetes was observed both in the Netherlands (Balvers et al., 2021) and Nigeria (Doumatey et al., 2020). *Anaerostipes* is associated with improved β-cell function and insulin efficiency (Diener et al., 2021). Taken together, *A. hadrus* may play a pivotal role in SCFA catabolism in the NDO microbiome community, reducing the risk of T2D in obesity in Mongolians.

In conclusion, this study provided the insight into the role of the gut microbiome in the prevention of T2D associated with obesity in Mongolians. Different microbial community was observed in obese subjects in Mongolia depending on the presence or absence of diabetes. Gut microbial bacteria such as *Prevotella*, *Faecalibacterium*, and *Anaerostipes*, as well as metabolites such as TUDCA, acetate, and total SCFA, were considered to play a foundational role in preventing the onset of diabetes, while certain disease-related bacteria potentially contribute to the onset of diabetes. However, this study has some limitations. As the number of subjects and sampling regions were limited, the generalization of the results of this study is restricted. It should be also noted that there are a number of hidden factors associated with the development of T2D other than the microbiome factors. On the other hand, even after adjusting for potential confounding variables, the elevated levels of acetate and total SCFA could have a substantial preventative impact on the development of T2D; this might be achieved by re-establishing the gut microbiome, with *Anaerostipes hadrus* as a keystone species.

## Data availability statement

The datasets presented in this study can be found in online repositories. The names of the repository/repositories and accession number(s) can be found in the article/Supplementary material.

## Ethics statement

The studies involving humans were approved by Ethics Committees of the Faculty of Agriculture in Kyushu University (Approval No. 107) and Committee of the Ministry of Health of Mongolia (Approval No. 78). The studies were conducted in accordance with the local legislation and institutional requirements. The participants provided their written informed consent to participate in this study.

## Author contributions

AS: Writing – original draft, Methodology, Investigation, Funding acquisition, Formal analysis. TL: Writing – original draft, Investigation. RM: Writing – original draft, Supervision, Investigation. PT: Supervision, Writing – review & editing. DJ: Writing – original draft, Investigation. CP: Writing – original draft, Investigation. SS: Writing – original draft, Investigation. BC: Writing – original draft, Investigation. BN: Writing – original draft, Investigation. YL: Writing – review & editing, Supervision. SD: Writing – review & editing, Supervision. JN: Writing – review & editing, Supervision, Project administration, Methodology, Funding acquisition, Formal analysis, Conceptualization.
