# Supplementary material for: Gut microbiome signatures associated with type 2 diabetes in obesity in Mongolia
Source: Front Microbiol. 2024 Jun 25;15:1355396. doi: 10.3389/fmicb.2024.1355396 (PMC11231203; doi:10.3389/fmicb.2024.1355396)
Supplement: Supplementary file 1 [file Image_1.pdf]

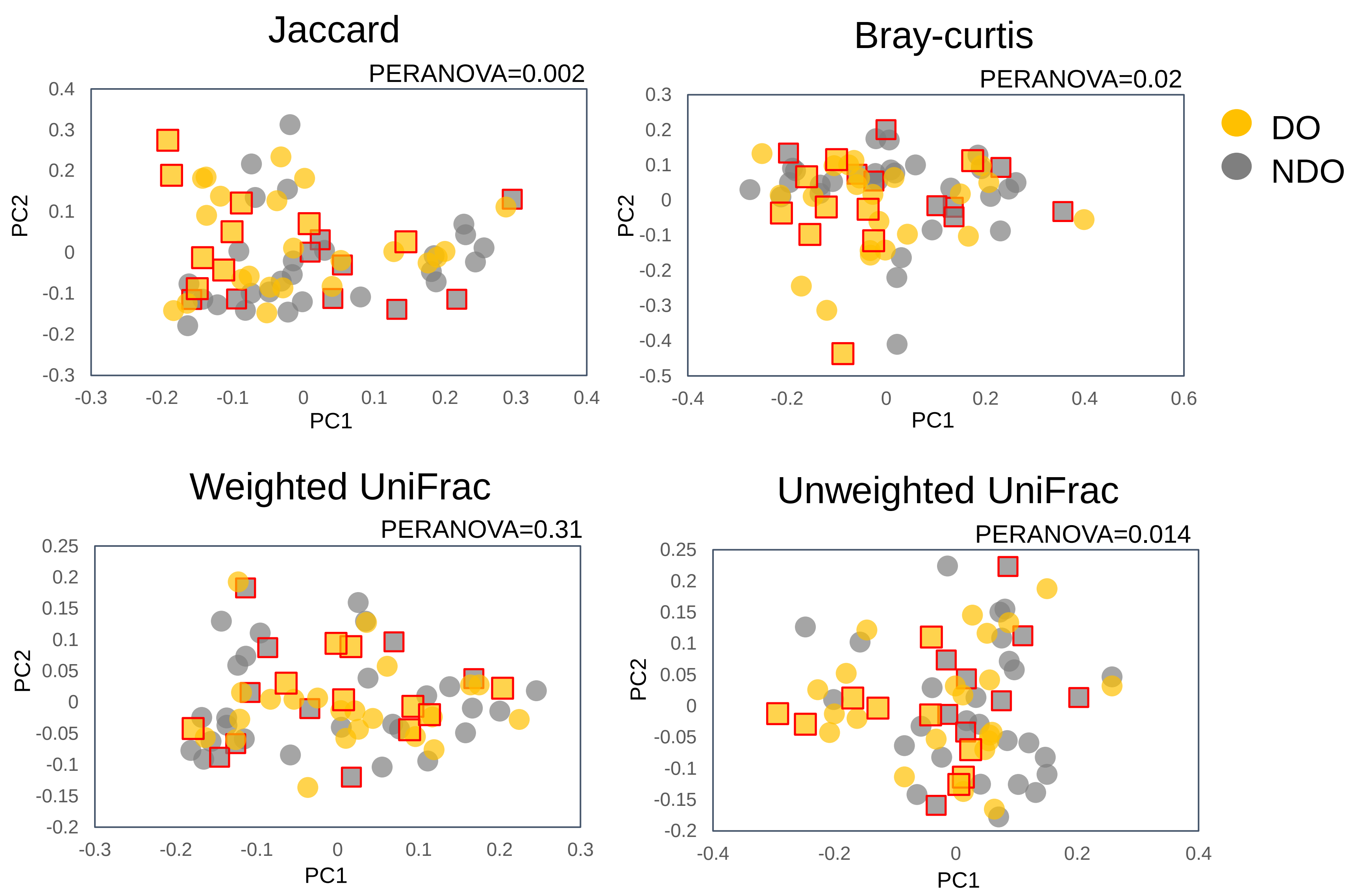

Supplementary Figure S1. Principal coordinates analysis (PCoA) plot of the 66 Mongolian samples based on the Jaccard, Bray-Curtis, weighted UniFrac, and unweighted UniFrac distance. Red squares show the samples selected for whole metagenomic analysis. Statistical significance of difference between the diabetic obese (DO) and nondiabetic obese (NDO) groups was assessed by PERANOVA (pairwise adonis).

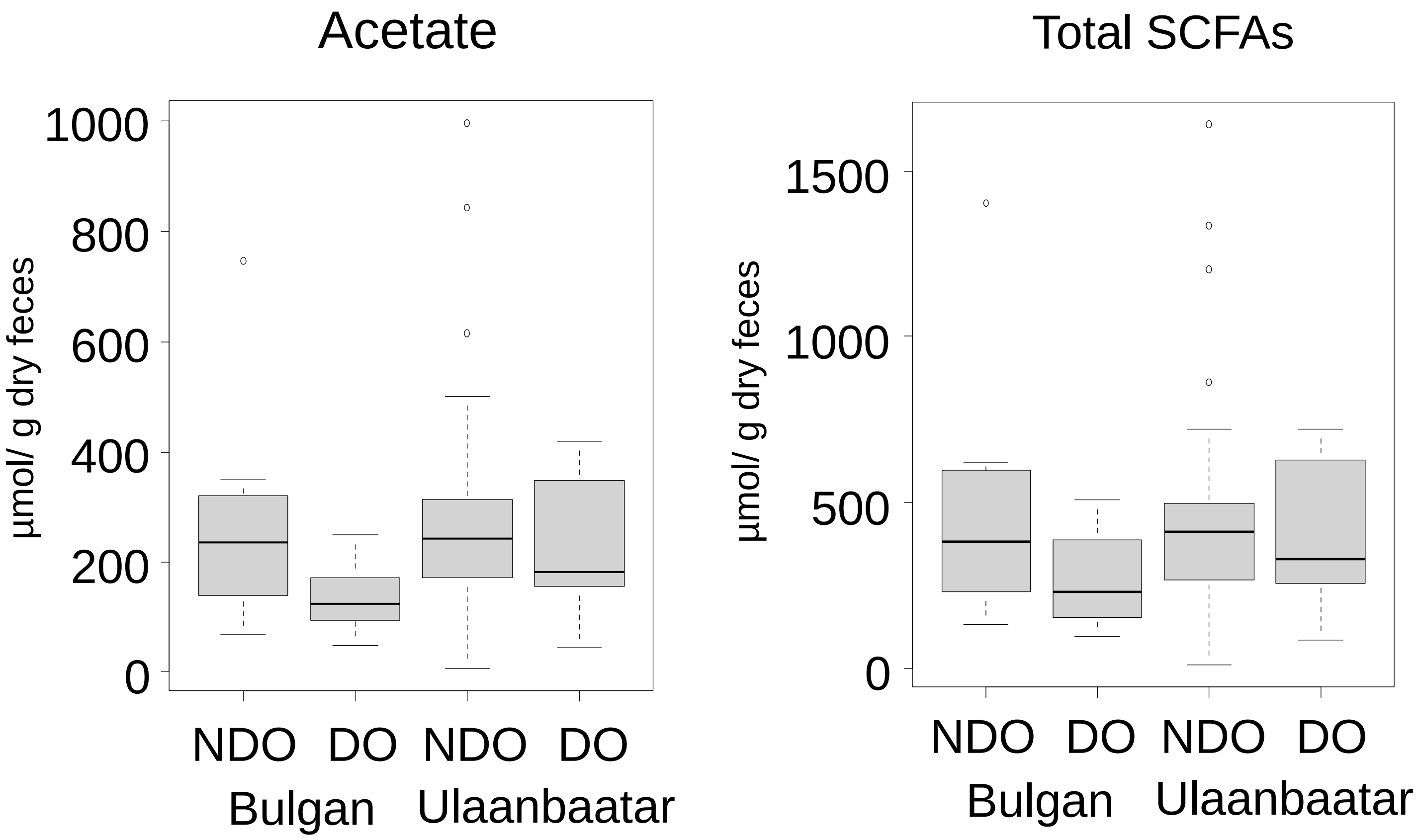

Supplementary Figure S2. Distribution of the levels of acetate and total short-chain fatty acids (SCFAs) in the feces of the diabetic obese (DO) and nondiabetic obese (NDO) groups in Ulaanbaatar and Bulgan. The box plot illustrates these levels in the DO and NDO groups; statistical significance was evaluated using the Wilcoxon rank-sum test. *, $0.05 < p < 0.1$, **, $p \leq 0.05$.

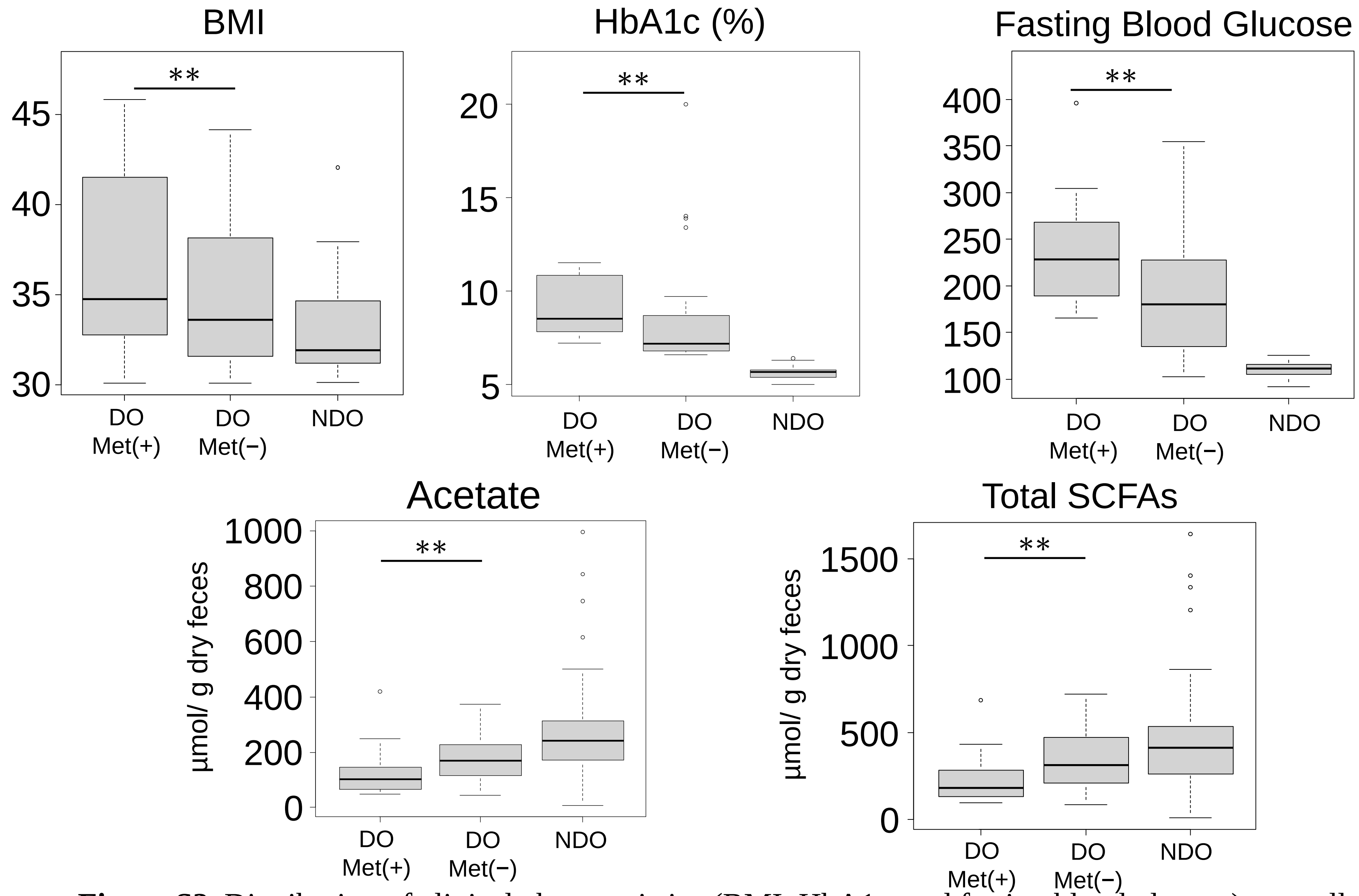

Supplementary Figure S3. Distribution of clinical characteristics (BMI, HbA1c, and fasting blood glucose), as well as the levels of acetate and total short-chain fatty acids (SCFAs) among three groups: diabetic obese group receiving metformin treatment (DO-Met(+)), diabetic obese group not receiving metformin treatment (DO-Met(−)), and nondiabetic obese (NDO) groups. Statistical significance between DO-Met(+) and DO-Met(−) was evaluated using the Wilcoxon rank-sum test. *, $0.05 < p < 0.1$, **, $p \leq 0.05$.
